# Structures of Two Human Astrovirus Capsid/Neutralizing Antibody Complexes Reveal Distinct Epitopes and Inhibition of Virus Attachment to Cells

**DOI:** 10.1128/JVI.01415-21

**Published:** 2022-01-12

**Authors:** Lena Ricemeyer, Nayeli Aguilar-Hernández, Tomás López, Rafaela Espinosa, Sarah Lanning, Santanu Mukherjee, Carolina Cuellar, Susana López, Carlos F. Arias, Rebecca M. DuBois

**Affiliations:** a Department of Biomolecular Engineering, University of California Santa Cruz, Santa Cruz, California, USA; b Departamento de Genética del Desarrollo y Fisiología Molecular, Instituto de Biotecnología, Universidad Nacional Autónoma de México, Cuernavaca, Morelos, Mexico; St. Jude Children's Research Hospital

**Keywords:** astrovirus, capsid, neutralizing antibodies, protein structure-function

## Abstract

Human astrovirus is an important cause of viral gastroenteritis worldwide. Young children, the elderly, and the immunocompromised are especially at risk for contracting severe disease. However, no vaccines exist to combat human astrovirus infection. Evidence points to the importance of antibodies in protecting healthy adults from reinfection. To develop an effective subunit vaccine that broadly protects against diverse astrovirus serotypes, we must understand how neutralizing antibodies target the capsid surface at the molecular level. Here, we report the structures of the human astrovirus capsid spike domain bound to two neutralizing monoclonal antibodies. These antibodies bind two distinct conformational epitopes on the spike surface. We add to existing evidence that the human astrovirus capsid spike contains a receptor-binding domain and demonstrate that both antibodies neutralize human astrovirus by blocking virus attachment to host cells. We identify patches of conserved amino acids which overlap or border the antibody epitopes and may constitute a receptor-binding site. Our findings provide a basis for developing therapies to prevent and treat human astrovirus gastroenteritis.

**IMPORTANCE** Human astroviruses infect nearly every person in the world during childhood and cause diarrhea, vomiting, and fever. Despite the prevalence of this virus, little is known about how antibodies block astrovirus infection. Here, we determined the crystal structures of the astrovirus capsid protein in complex with two virus-neutralizing antibodies. We show that the antibodies bind to two distinct sites on the capsid spike domain, however, both antibodies block virus attachment to human cells. Importantly, our findings support the use of the human astrovirus capsid spike as an antigen in a subunit-based vaccine to prevent astrovirus disease.

## INTRODUCTION

Astroviruses are a distinct family of small, nonenveloped RNA viruses that infect mammalian and avian species (1). Members of the *Avastrovirus* genus have been associated with a variety of disease manifestations, growth defects, and mortality in poultry (2). Members of the *Mamastrovirus* genus cause infections in humans and a wide range of other mammals, indicating their potential for a zoonotic disease transmission and the emergence of new astrovirus strains that could threaten human health (3). Within *Mamastrovirus*, human astroviruses (HAstVs) are classified into eight canonical serotypes (HAstV1-8), where serotype 1 is the most prevalent globally (4–7). HAstV is a leading worldwide cause of viral gastroenteritis but remains one of the most inadequately understood enteric viruses (8). Young children, the elderly, and the immunocompromised are at particular risk for astrovirus infection, especially in developing countries (9–16). The United States alone reports approximately 3.9 million cases of HAstV gastroenteritis each year (17), and children under 12 months may require hospitalization (18). While HAstV infection accounts for an estimated 2% to 9% of all acute nonbacterial gastroenteritis in healthy children worldwide (19), studies found that HAstV also causes persistent infections that spread easily in pediatric oncology wards (20, 21). In addition to the canonical serotypes causing gastroenteritis, the highly divergent MLB and VA clades have recently been associated with neurological complications such as encephalitis in immunocompromised and immunocompetent individuals, demonstrating that astroviruses not only infect cells in the gastrointestinal tract but also have systemic potential (22–25). However, no vaccine has been developed to prevent human astrovirus infection. Additionally, while a few existing antiviral therapies have been shown to be effective against multiple astrovirus serotypes *in vitro* and in the turkey poult animal model (26, 27), their use for treating astrovirus infection in humans has yet to be reported.

A growing body of evidence underlines the importance of antibodies in protecting healthy adults from infection. First, human astrovirus infection is rare in adults, indicating that a protective adaptive immune response conveying lifelong immunity develops during childhood (28). In fact, more than 75% of healthy adults have anti-HAstV antibodies targeting at least one of the eight classical serotypes (7, 28), and seroprevalence rates increase with age (5). Second, clinical studies of healthy volunteers determined that more severe disease is correlated with a lack of anti-HAstV antibodies (29, 30). Finally, immunoglobulin replacement therapy helped an immunocompromised patient recover from a persistent HAstV infection (31). Together, these findings suggest that the incidence of HAstV gastroenteritis is likely underappreciated. In addition, these observations reveal that the adaptive immune response plays a crucial role in shielding an individual from HAstV disease. Accordingly, we anticipate that a vaccine eliciting protective antibodies will reduce HAstV infection in vulnerable populations. However, rational design of subunit vaccine immunogens or antiviral therapies relies on an understanding of the sites at which neutralizing antibodies bind human astrovirus and on insight into viral defenses against antibody neutralization.

HAstV particles contain a 6- to 7-kb, positive-sense, single-stranded RNA genome surrounded by an ∼35 nm nonenveloped capsid protein shell. The genome’s three open reading frames (ORFs) encode the nonstructural polyproteins (ORF1a and ORF1b) and the multidomain capsid protein (ORF2) (32, 33). This capsid protein contains a highly basic N-terminal region, a core domain, a spike domain, and a C-terminal acidic region (34). During maturation, HAstV capsid proteins undergo a series of intra- and extracellular proteolytic cleavages that are required for infectivity (35–38). Our lab and others have solved the crystal structure of the capsid core domain, which forms the T = 3 icosahedral shell that encapsidates the viral RNA genome (39, 40). Our lab and others have also solved the crystal structure of the capsid spike domain, which forms the 30 dimeric spike projections on the surface of the mature virus particle (39, 41, 42).

While the structural characterization of the human astrovirus capsid has made these advancements, further study is needed to truly understand the functional sites on the HAstV capsid, including the location of the receptor-binding site(s), the epitopes where neutralizing antibodies bind, and the identity of the unknown host cell receptor(s). Only one neutralizing epitope, located on the capsid spike domain, has been defined by X-ray crystallography of an antibody/spike complex (42). This study provided evidence that the spike is a receptor-binding domain and that this antibody, PL-2, neutralizes HAstV2 by obstructing a receptor-binding site on the spike. Further study has shown that while both the core and spike domains of the HAstV capsid are immunogenic, only the spike domain elicits antibodies which neutralize virus infectivity (43). Indeed, the neutralizing monoclonal antibodies against HAstV for which neutralization mechanisms have been described all target the spike domain (43–45).

To better understand the HAstV antigenic structure and the mechanistic basis of virus neutralization, we report the structures of the HAstV8 capsid spike (Spike 8) bound to two recently discovered HAstV8-neutralizing monoclonal antibodies, 3E8 and 2D9 (43), at resolutions of 2.05 Å and 2.65 Å, respectively. These structures reveal two separate, nonoverlapping epitopes that each contain conserved amino acids. We determine that both 3E8 and 2D9 neutralize HAstV8 by blocking attachment to cells. Our studies lay a foundation for the mechanistic understanding of HAstV infectivity and provide a blueprint for the design of vaccine immunogens that induce neutralizing antibodies against all classical HAstV serotypes.

## RESULTS

### Neutralizing antibodies 3E8 and 2D9 have high affinity for the HAstV8 capsid spike.

**FIG 1:**
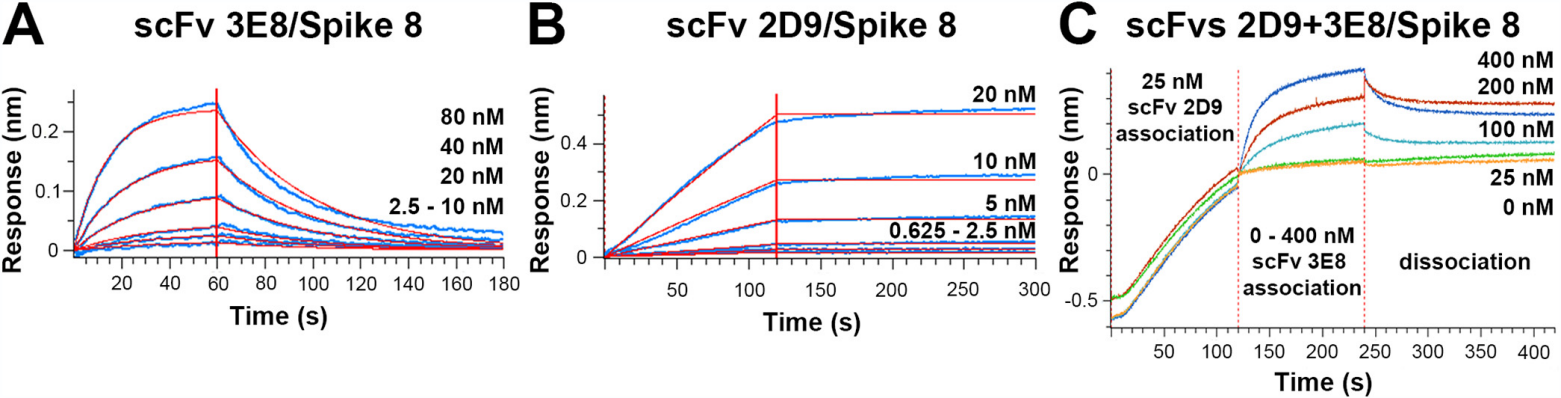
scFv 3E8 and scFv 2D9 bind Spike 8 with high affinity. Spike 8 was loaded onto biosensor tips, then placed into serial dilutions of each scFv to allow the association of antibody to spike, and finally placed into buffer to allow the dissociation of the antibody/Spike 8 interaction. (A) Affinity determination for the scFv 3E8/Spike 8 interaction. (B) Affinity determination for the scFv 2D9/Spike 8 interaction. (C) Competition BLI assay showing simultaneous binding of scFvs 3E8 and 2D9 to Spike 8. Data shown in panels A and B are from one representative experiment of triplicate assays.

Previously, mouse monoclonal antibodies (MAbs) 3E8 and 2D9 were raised against recombinant Spike 8 in mice and were shown to neutralize the infectivity of HAstV8 in human colon adenocarcinoma cells (Caco-2 cells), the standard cell line for HAstV propagation (43). We first examined the affinities of purified recombinant antibodies 3E8 and 2D9 for purified recombinant Spike 8. To assess and quantify binding, we performed biolayer interferometry (BLI) experiments. We used single-chain variable fragment (scFv) antibody constructs in these kinetic studies to eliminate the potential complications of avidity with bivalent antibody and dimeric Spike 8. After a baseline measurement, Spike 8 fused to a 10× histidine tag was loaded onto Anti-Penta-His (HIS1K) biosensors which were subsequently submerged in 1:2 serial dilutions of scFv to measure on-rates and then submerged in buffer to measure off-rates. We found that scFv 3E8 and scFv 2D9 bind to Spike 8 in a dose-dependent manner with high-affinity dissociation constants (K_D_) of 40.03 ± 2.37 nM and 2.45 ± 0.26 pM, respectively (Fig. 1A and B and Table 1). From these results, we conclude that the immunization of mice with Spike 8 antigen produces high-affinity HAstV8-neutralizing antibodies. These studies support the development of a subunit vaccine for human astrovirus using recombinant spikes as immunogens.

**TABLE 1:** scFv 3E8 and scFv 2D9 bind Spike 8 with high affinity

Antibody/spike complex	*K_D_*^*a*^ ± σ	*R* ^2^	χ^2^
scFv 3E8/Spike 8	40.03 ± 2.37 nM	0.9900	0.4435
scFv 2D9/Spike 8	2.45 ± 0.26 pM	0.9968	0.8963

*^a^K_D_* = equilibrium dissociation constant.

Next, we performed a competition BLI experiment to determine whether 3E8 and 2D9 compete for the same target on Spike 8 or whether they can bind Spike 8 simultaneously. We loaded Spike 8 onto the biosensors and first allowed scFv 2D9, which has the stronger affinity, to bind it. We associated the higher-affinity scFv 2D9 first to ensure that it would not be displaced upon the association of the weaker-affinity scFv 3E8. We then dipped into dilutions of scFv 3E8 and observed an additional association signal, suggesting that 3E8 and 2D9 can bind Spike 8 at the same time and therefore at different epitopes (Fig. 1C).

This result is in accordance with those of previous escape mutation studies, in which HAstV8 escape mutants were generated using mouse MAbs 3E8 and 2D9 (43). Virus was cultured with low concentrations of one mouse MAb. Under selective pressure, the virus mutated to escape MAb neutralization. After sequencing, the viral variant able to escape neutralization by mouse MAb 3E8 contained the mutation Y464H in the Spike 8 sequence. In contrast, the viral variant able to escape neutralization by mouse MAb 2D9 contained the mutation D597Y in the Spike 8 sequence. Both of these mutations map to the surface of Spike 8, but are ∼30 Å distant from each other in space, hinting at nonoverlapping epitopes. In addition, MAb 3E8 still effectively neutralized the MAb 2D9 viral escape mutant, and vice versa. These results suggest that the Spike 8 epitopes recognized by MAb 3E8 and MAb 2D9 are situated in different locations on the Spike 8 surface, but they do not provide a complete picture of the epitope footprint(s) present on Spike 8.

### scFv 3E8 binds Spike 8 at a known epitope.

**FIG 2:**
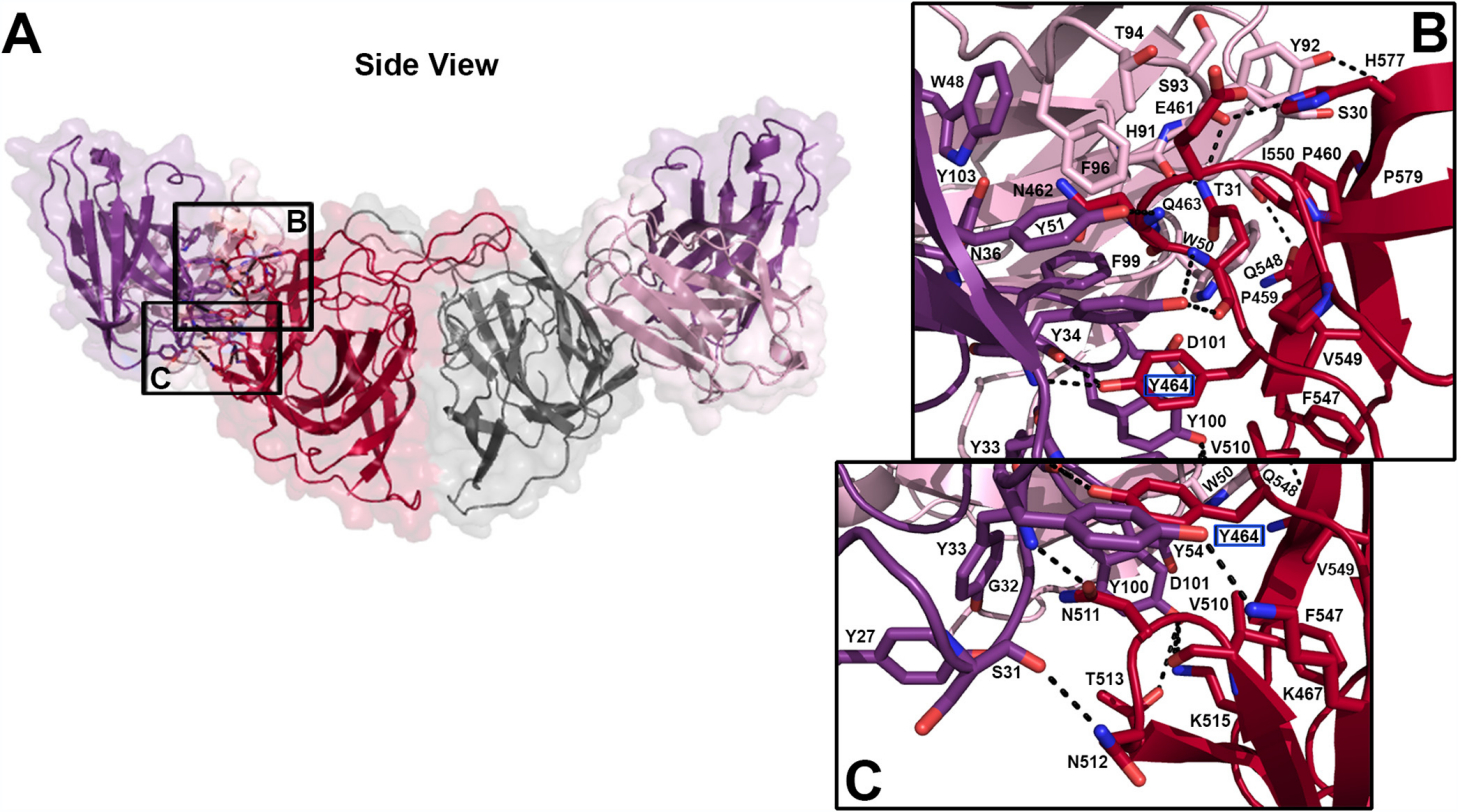
Intermolecular interactions at the scFv 3E8/Spike 8 interface. (A) Semitransparent surface representation of the scFv 3E8/Spike 8 complex. The Spike 8 dimer is colored red and gray, the scFv 3E8 kappa chain is colored pale purple, and the scFv 3E8 heavy chain is colored dark purple. Primary regions of interaction are highlighted by black boxes with labels corresponding to panels B and C. Dashed lines represent hydrogen bonds. Escape mutant residue Y464 is indicated by a blue box around the residue name. (B) Interactions of 3E8 complementarity-determining regions (CDRs) K3, H1, H2, and H3 with Spike 8 Loop 1. Interactions of 3E8 CDRs K1, K2, K3, and H3 with the Spike 8 β8 strand. Interactions of CDRs K1 and K3 with the Spike 8 β9 strand. (C) Interactions of 3E8 CDRs H1 and H3 with Spike 8 β5-β6 strands.

Previously, we had solved the structure of a HAstV2-specific neutralizing antibody, PL-2, in complex with the HAstV2 capsid spike (Spike 2) (42). This PL-2 antibody mainly contacts Spike 2 Loop 1, and the epitope contains residues directly adjacent to 3E8 escape mutant Y464. Therefore, PL-2 and 3E8 may target a similar epitope. To define the MAb 3E8 binding site on Spike 8, we cocrystallized and solved the structure of the scFv 3E8/Spike 8 complex to a resolution of 2.05 Å (Fig. 2 and Table 2). For both structures in this text, we attempted crystallization of complexes with either scFv or recombinant antigen-binding fragment (Fab) constructs, but only obtained high-resolution crystal diffraction data from scFv/Spike 8 complexes. The structure confirms size exclusion chromatography data demonstrating that scFv 3E8 binds to Spike 8 at a 2:2 ratio: one scFv 3E8 molecule makes contact with one protomer of the Spike 8 dimer, and a second scFv 3E8 molecule binds the same epitope on the other Spike 8 protomer (Fig. 2). We observed a conformation-dependent tertiary epitope on each Spike 8 protomer which overlaps with the PL-2-targeted epitope (discussed further below). Four segments of amino acids from one linear Spike 8 molecule comprise the three-dimensional 3E8-targeted epitope.

**TABLE 2:** Data collection and refinement statistics for scFv 3E8/Spike 8 and scFv 2D9/Spike 8^*a*^

Characteristic	scFv 3E8/Spike 8	scFv 2D9/Spike 8
PDB code	7RK1	7RK2
		
Data collection		
Space group	P 1 21 1	P 21 21 21
Cell dimensions		
*a*, *b*, *c* (Å)	48.83, 80.43, 117.21	49.59, 97.34, 208.31
α, β, γ (°)	90.00, 90.67, 90.00	90.00, 90.00, 90.00
Resolution (Å)	80.43–2.05 (2.11–2.05)	208.31–2.65 (2.78–2.65)
*R*_sym_ or *R*_merge_	0.184 (1.268)	0.451 (3.877)
*I*/σ*I*	7.2 (2.2)	16.0 (2.0)
Completeness (%)	97.9 (94.8)	100 (99.8)
Redundancy	6.1 (5.3)	76.5 (66.6)
CC_1/2_	0.992 (0.602)	0.998 (0.692)
		
Refinement		
No. of reflections	55,630	30,211
Resolution (Å)	66.32–2.05	104.15–2.65
R_work_/R_free_	0.223/0.238	0.218/0.247
No. of atoms	7,378	7,105
Protein	7,043	6,955
Ligands	0	0
Water	335	150
B factors	32.18	40.05
Protein	32.13	40.15
Ligands	NA	N/A
Water	33.32	35.52
RMSD		
Bond lengths (Å)	0.012	0.013
Bond angles (°)	1.70	1.49
Ramachandran statistics		
Favored (%)	97.5	97.5
Allowed (%)	2.5	2.5
Outliers (%)	0	0

*^a^*Data from one crystal were used for each structure determination. Values in parentheses are for the highest-resolution shell. RMSD, root-mean-square-deviation.

All six complementarity-determining regions (CDRs) from the kappa (K) and heavy (H) chains of each scFv 3E8 interact with one protomer of Spike 8 (Fig. 2). The interface is defined by several contact points: (i) scFv 3E8 CDR H2 contacts Spike 8 Loop 1 residues P459, P460, E461, N462, and K467, while scFv 3E8 CDR H1 contacts N462, Q463, and Y464. CDRs K3 and H3 also contact Spike 8 Q463 and Y464, respectively (Fig. 2B). (ii) scFv 3E8 CDR H3 contacts Spike 8 β5-β6 residues V510, N511, T513, and K515, and CDR H1 contacts N511 and N512 (Fig. 2C). (iii) scFv 3E8 CDR H3 contacts Spike 8 β8 residue F547, CDRs K1 and K2 contact Q548, and CDRs K2 and K3 contact V549 and I550 (Fig. 2B). (iv) scFv 3E8 CDR K3 contacts Spike 8 β9 residue H577, and CDRs K1 and K3 contact P579 (Fig. 2B). scFv 3E8 binds Spike 8 through a large network of hydrogen bonds, electrostatic bonds, and van der Waals interactions. Many of the amino acids in the 3E8 epitope are unique to serotype 8, consistent with virus neutralization data demonstrating that 3E8 is serotype-specific (43). However, three residues, K515, F547, and V549, are strictly conserved across all eight serotypes, while P460 and K467 are mostly conserved. Spike 8 residue Y464, the location of the Y464H escape mutant, is buried by scFv 3E8 binding (Fig. 2B and C). Compared to the wild-type tyrosine, the side chain of the histidine mutant may be too short to form hydrogen bonds with either the backbone nitrogen of scFv 3E8 CDR H1 residue Y34 and/or the backbone carbonyl oxygen of scFv 3E8 CDR H3 residue F99, and this would thereby affect antibody binding. Moreover, Y464 makes pi-stacking interactions with scFv 3E8 CDR H1 residue Y34, CDR H2 residue Y54, and CDR H3 residues F99 and Y100, all within 3 to 5 Å; a mutation to histidine may diminish these interactions. The discovery that Spike 8 residue Y464 is part of the epitope signifies that the escape mutation studies accurately indicated the epitope’s approximate location. However, only the structure of the entire complex revealed the full extent of the scFv 3E8/Spike 8 binding interface.

### scFv 2D9 binds Spike 8 at a novel epitope.

**FIG 3:**
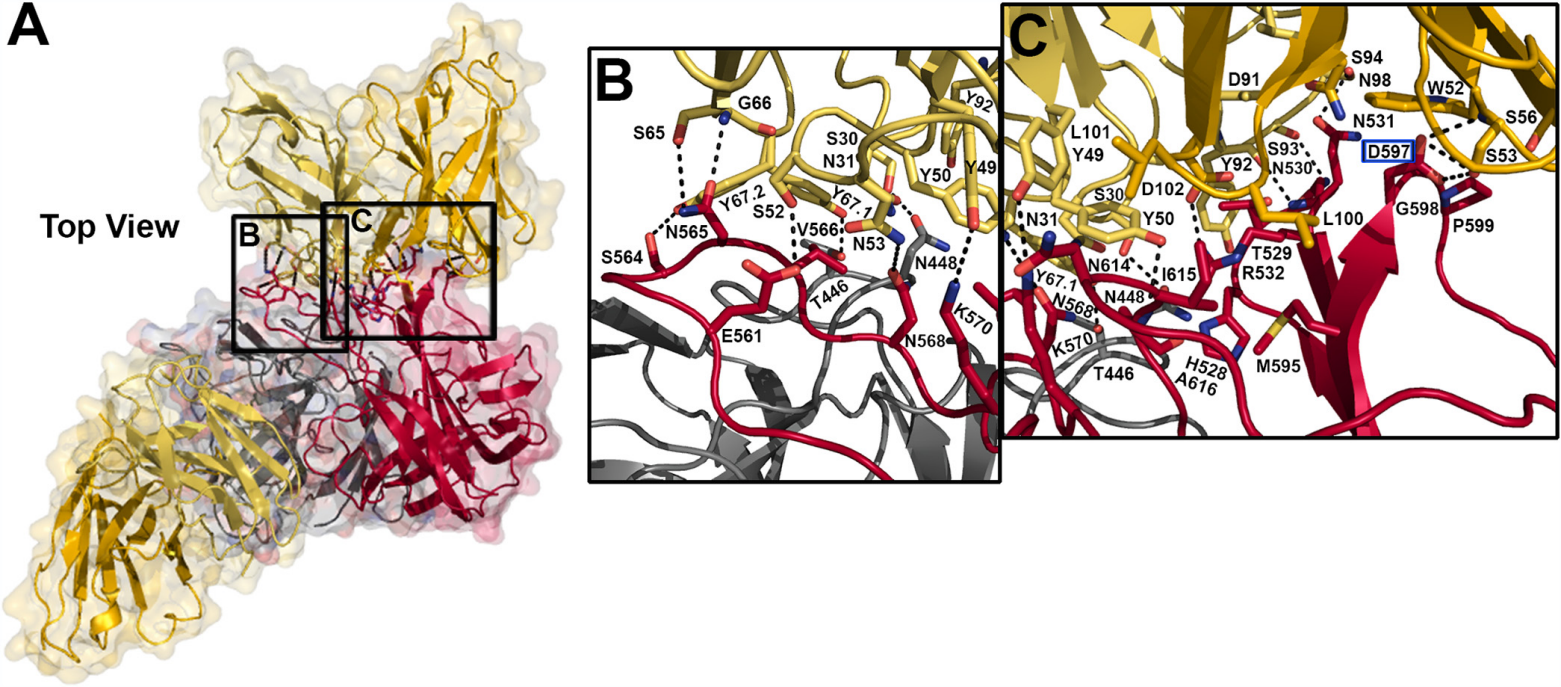
Intermolecular interactions at the scFv 2D9/Spike 8 interface. (A) Semitransparent surface representation of the scFv 2D9/Spike 8 complex. The Spike 8 dimer is colored red and gray, the scFv 2D9 kappa chain is colored pale yellow, and the scFv 2D9 heavy chain is colored gold. Primary regions of interaction are highlighted by black boxes with labels corresponding to panels B and C. Dashed lines represent hydrogen bonds. Escape mutant residue D597 is indicated by a blue box around the residue name. (B) Interactions of 2D9 complementarity-determining regions (CDRs) K1 and K2 with Spike 8 Loop 1 (gray protomer) and Spike 8 Loop 3 (red protomer). (C) Interactions of 2D9 CDRs K2, K3, H2, and H3 with Spike 8 β6-β7 and β10 strands, as well as with Loop 4 (all on the red protomer).

To fully characterize the MAb 2D9 binding site on Spike 8 and compare it to the 3E8 and PL-2 binding sites, we next cocrystallized and solved the structure of the scFv 2D9/Spike 8 complex to a resolution of 2.65 Å (Fig. 3 and Table 2). The structure confirms size exclusion chromatography data which demonstrate that scFv 2D9 binds to Spike 8 at a 2:2 ratio, the same ratio as for scFv 3E8/Spike 8 and scFv PL-2/Spike 2. In contrast to the 3E8 tertiary epitope, we find that scFv 2D9 binds to a conformation-dependent quaternary epitope across the Spike 8 dimer interface which is fully distinct from the epitope targeted by 3E8 and PL-2. Five patches of amino acids from two linear Spike 8 molecules come together in the three-dimensional structure.

Five complementarity-determining regions (CDRs) from the kappa (K) and heavy (H) chains of each scFv 2D9 contact Spike 8 (Fig. 3). The interface is characterized by several interactions: (i) scFv 2D9 kappa chain residue Y67 near CDR K1, which exhibits electron density for two conformations in the structure, contacts T446 with Orientation 1 (Y67.1) in Loop 1 of one Spike 8 protomer. In addition, scFv 2D9 CDR K1 contacts N448 on that protomer (Fig. 3B). The remaining interactions take place on the other Spike 8 protomer, indicating a quaternary epitope. (ii) scFv 2D9 CDR K3 contacts Spike 8 residues H528, T529, N530, and N531 at the β6-β7 junction. CDR H3 also contacts T529, and both CDR H3 and CDR K2 contact R532. (Fig. 3C). (iii) scFv 2D9 CDR K2 contacts Spike 8 E561, scFv 2D9 kappa chain Y67 Orientation 2 (Y67.2) contacts Spike 8 S564, and scFv 2D9 kappa chain S65 and G66 both contact N565. scFv 2D9 kappa chain Y67.1 and CDR K1 bury Spike 8 V566. CDR K2 contacts Spike 8 N568 and K570. These Spike 8 residues are all in Loop 3 (Fig. 3B). (iv) scFv 2D9 CDR H3 contacts Spike 8 M595 in the β10 strand. scFv 2D9 CDR H2 contacts Spike 8 β10 residues D597, G598, and P599 (Fig. 3C). (v) scFv 2D9 CDR K2 contacts Spike 8 Loop 4 residues N614 and A616. CDR H3 buries Spike 8 I615 (Fig. 3C). Similarly to scFv 3E8, scFv 2D9 relies on a large system of hydrogen bonds, electrostatic bonds, and van der Waals interactions to bind to a quaternary epitope on the surface of the Spike 8 dimer. Notably, Spike 8 residue D597, which mutated to Y during escape mutant studies, is buried by scFv 2D9 binding (Fig. 3C). The mutation of aspartate to the larger tyrosine likely caused steric clashing with scFv 2D9 CDR H2 residue S53, completely prohibiting antibody binding.

**FIG 4:**
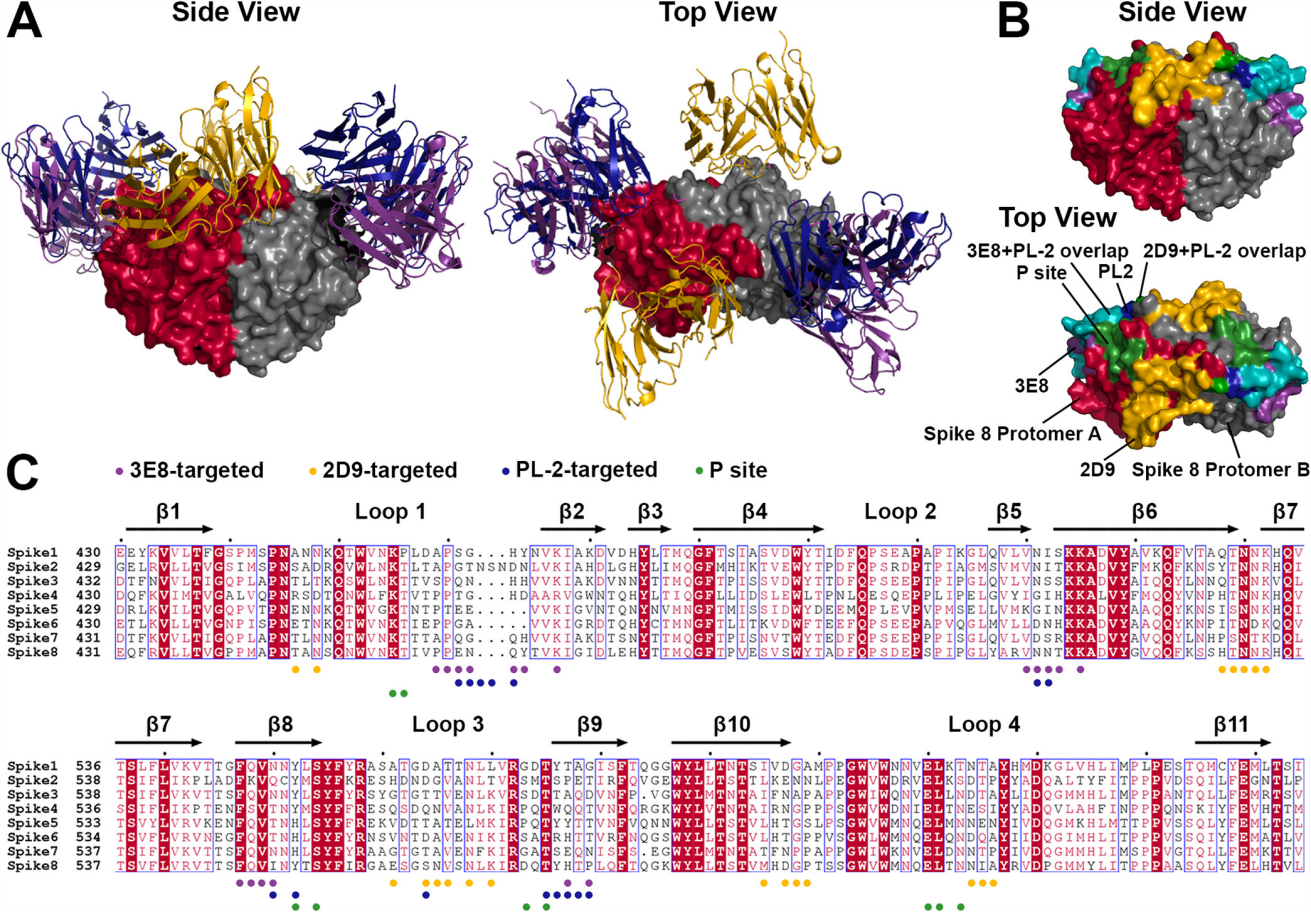
Comparison of epitopes for 3E8/Spike 8, 2D9/Spike 8, and PL-2/Spike 2 in relation to the P site. (A) Structure of scFv 3E8 (purple), scFv 2D9 (yellow), and scFv PL-2 (blue) bound to spike (red and gray), viewed from the side and from the top. (B) Epitope footprints on spike. The spike dimer and the footprints for 3E8, 2D9, and PL-2 are colored as in panel A. The eight-residue 3E8 + PL-2 overlap is colored teal. The single-residue 2D9 + PL-2 overlap is colored grass green. The P site is colored forest green. (C) Sequence alignment of the capsid spike domains of HAstV1-8. Conserved, strongly similar, weakly similar, and nonconserved amino acids are colored on a gradient, where darker color represents more conservation. Alignments were done with Clustal Omega (46), and mapping of conservation onto the structure was performed with the online ESPript server (47). Dots below the sequence alignment indicate residues in the epitopes targeted by 3E8 (purple), 2D9 (yellow), and PL-2 (blue), as well as residues in the P site (green).

While some amino acids in the epitope bound by scFv 2D9 are unique to HAstV8, the epitope also overlaps or abuts several patches of amino acids which are highly conserved between serotypes 1 to 8, suggesting that scFv 2D9 may block an important functional site on the Spike 8 surface. Interestingly, the epitope includes all four amino acids (T529, N530, N531, and R532) of an exposed, flexible β-turn knob which is very well conserved among all serotypes and hypothesized to interact with other viral or host proteins (Fig. 3C and Fig. 4C) (41). Critically, there is no overlap in the Spike 8 residues targeted by scFv 3E8 and scFv 2D9, indicating two separate and distinct epitopes on the human astrovirus capsid spike surface (Fig. 4A and B).

### Antibodies 3E8, 2D9, and PL-2 target epitopes near the P site.

The human astrovirus host cell receptor is unidentified. In addition, the receptor-binding site(s) on the HAstV capsid surface is unknown. Because 3E8, 2D9, and PL-2 all inhibit virus attachment to cells (discussed further below) (42), it is likely that each of them prevents virus access to the receptor. Several putative receptor-binding sites on the spike surface have been proposed (41). Among them is the conserved P site, located in a shallow groove on top of the spike domain (Fig. 4B and C). The P site has many hydrophilic residues with side chains exposed to solvent, making it highly accessible to potential cell receptors without steric hindrance. While Spike 8 residues in the 3E8, 2D9, and PL-2 epitopes mostly do not overlap with residues in the P site, the former do appear to frame the P sites on each Spike 8 protomer (Fig. 4B). If the P site plays a role in virus attachment to the host cell receptor, its placement between the epitopes may prevent the virus from accessing the receptor when any of these three antibodies bind.

Figure 4C presents a sequence alignment of the spikes from all eight classical HAstV serotypes and indicates residues in the P site and in the epitopes targeted by 3E8, 2D9, and PL-2. Eight of these residues are located in epitopes targeted by both 3E8 and PL-2 (although N511 is the only one with sequence identity between Spike 2 and Spike 8), revealing this region’s particular vulnerability to antibody binding across HAstV serotypes. While there is no overlap between the 3E8 and 2D9 epitopes, S564 (D566 in Spike 2) is found in both the 2D9 and PL-2 epitopes. Sequence conservation may indicate that 3E8, 2D9, and PL-2 obstruct a conserved functional site on the virus surface, either directly or by proximity. In the next section, we demonstrate that 3E8 and 2D9 neutralize HAstV8 by the same mechanism despite targeting fully separate epitopes.

### Antibodies 3E8 and 2D9 block attachment of HAstV8 to Caco-2 cells.

**FIG 5:**
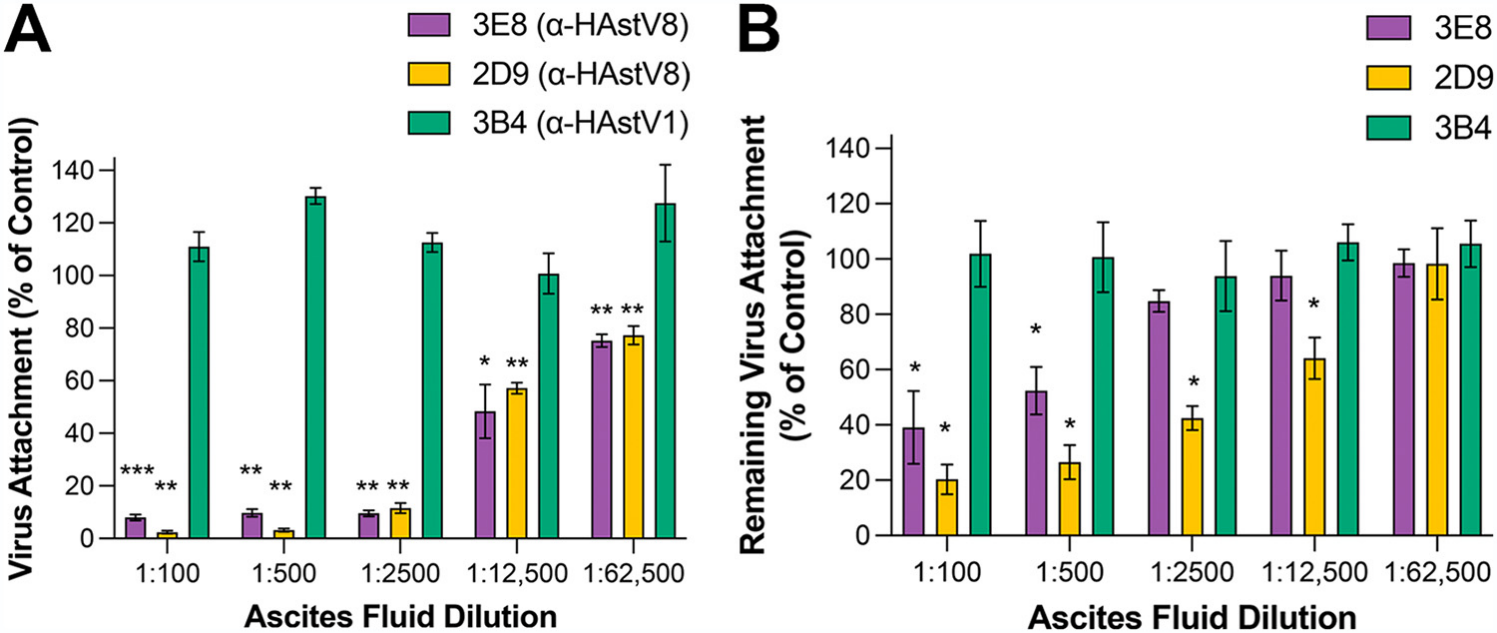
Antibodies 3E8 and 2D9 block virus attachment and detach the virus from Caco-2 cells. (A) 3E8 and 2D9 block virus attachment when HAstV8 is pre-incubated with the antibodies. (B) 3E8 and 2D9 detach HAstV8 from cells when the antibodies are added after the virus has attached. Experiments were performed on ice to prevent virus endocytosis. The arithmetic means and standard deviations are from four (attachment) or three (detachment) independent experiments, performed either in duplicate (3E8 and 2D9) or as single replicas (3B4), with * *P* < 0.05, ** *P* < 0.01, and *** *P* < 0.001.

Previously, we showed that MAbs 3E8 and 2D9 reduced virus infectivity (43), which decreased with increasing amounts of antibody. To determine the neutralization mechanisms of 3E8 and 2D9, we next explored whether these antibodies could block virus attachment to the surface of Caco-2 cells. Purified HAstV8 particles were pre-incubated with serial dilutions of the ascites fluids of either 3E8 or 2D9, and the virus-antibody mix was added to Caco-2 cell monolayers on ice. At this temperature, the virus can attach to the cell surface but does not enter the cell. The unbound virus was removed by washing, total RNA was extracted, and the viral genome was quantified by RT-qPCR. As shown in Fig. 5A, both antibodies were able to efficiently impede HAstV8 attachment; at a 1:2,500 dilution of the ascites fluids, they reduced virus binding by more than 90% compared to virus binding in the absence of antibody. As a negative control, we added MAb 3B4, which is specific for HAstV1 and has been previously shown not to neutralize HAstV8 infectivity; no reduction of virus binding was observed with this antibody. This result shows a direct correlation between inhibition of virus infectivity and inhibition of virus binding.

To confirm that MAbs 3E8 and 2D9 inhibit virus attachment directly and not through aggregation of virus particles, we evaluated their ability to detach the virus once it has bound to the cell surface. For this assay, purified HAstV8 was added to Caco-2 cells and incubated on ice. The unbound virus was removed by washing, and then serial 1:5 dilutions of the ascites fluids of 3E8, 2D9, or 3B4 were added to the cells and further incubated on ice. After this incubation, the cells were washed again, and viral RNA was quantified as described above. As shown in Fig. 5B, both MAbs 2D9 and 3E8 were able to detach the virus from the cell surface, although 2D9 did so more efficiently; presumably, these antibodies accomplished d by competing with the virus-receptor interaction. Negative control 3B4 was unable to detach the virus. Altogether, these results indicate that antibodies 3E8 and 2D9 neutralize virus infectivity by inhibiting the attachment of the virus to the cell surface.

### Antibodies 3E8 and 2D9 block attachment of GFP-Spike 8 to Caco-2 cells.

**FIG 6:**
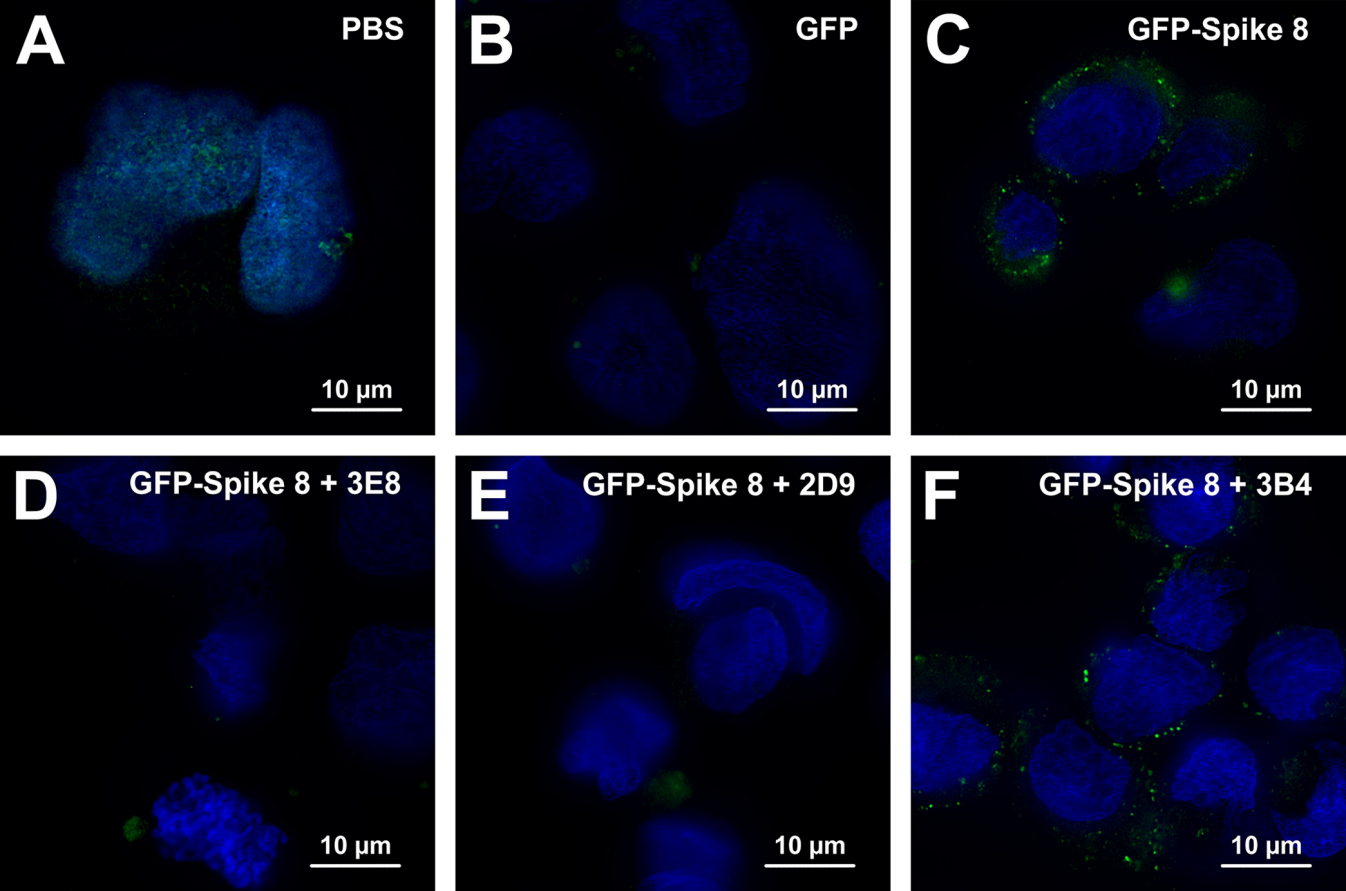
Antibodies 3E8 and 2D9 block GFP-Spike 8 attachment to Caco-2 cells. GFP fluorescence is green and Hoechst DNA stain is blue. (A) Caco-2 cells incubated with PBS show slight autofluorescence. (B) Caco-2 cells incubated with GFP alone show little to no nonspecific GFP binding. (C) Caco-2 cells incubated with GFP-Spike 8 show bright punctate patterns at or near the cell membranes, suggesting spike-specific attachment. (D) Fab 3E8 blocks GFP-Spike 8 attachment to Caco-2 cells. (E) Fab 2D9 blocks GFP-Spike 8 attachment to Caco-2 cells. (F) Fab 3B4 does not block GFP-Spike 8 attachment to Caco-2 cells. Images are representative of data from at least three independent experiments.

Finally, to test if the human astrovirus capsid spike alone is sufficient for cell attachment and to show that antibodies block this attachment, we examined both the capacity of a recombinantly expressed green fluorescent protein (GFP)-Spike 8 fusion protein to attach to Caco-2 cells and the ability of recombinant antigen-binding fragments (Fabs) 3E8 and 2D9 to prevent this attachment. We chose Fab constructs for this set of experiments to avoid the possible dimerization effect of MAbs and (occasionally) scFvs, which could potentially cause cross-linking issues with dimeric GFP-Spike 8. Caco-2 cells grown on glass coverslips were incubated with GFP alone, GFP-Spike 8 alone, or GFP-Spike 8 with Fab 3E8, 2D9, or 3B4. Cells were washed, fixed, and imaged by fluorescence microscopy. As shown in Fig. 6, Caco-2 cells incubated with phosphate-buffered saline (PBS) alone or with GFP alone had little to no green signal (Fig. 6A and B). In contrast, Caco-2 cells incubated with GFP-Spike 8 had bright green punctate patterns that appeared at or near the cell membranes (Fig. 6C). Furthermore, cells incubated with GFP-Spike 8 and either Fab 3E8 or Fab 2D9 had minimal puncta (Fig. 6D and E), suggesting that the Fabs specifically blocked Spike 8 attachment to cells. The negative control Fab 3B4 did not block attachment (Fig. 6F). These data reveal that the human astrovirus capsid spike alone is sufficient for cell attachment and that antibodies 3E8 and 2D9 are able to preclude virus attachment by specifically blocking the spike.

## DISCUSSION

Our findings uncover several key insights into the biology of human astrovirus and the antibody response to the human astrovirus capsid antigen. First, we have shown that immunization with recombinant capsid spike elicits high-affinity antibodies that neutralize the virus, making the spike a candidate antigen for a subunit vaccine. We have also discovered that there are at least two distinct epitopes on the HAstV capsid spike surface. Antibodies targeting either one of these epitopes neutralize the virus by inhibiting virus attachment to cells. There may be several possible explanations for this phenomenon. The receptor-binding site may be in between the two epitopes, such as in the proposed P site, so that the binding of an antibody to either epitope sterically hinders viral access to the receptor. The receptor-binding site may also be very large and therefore contain residues in both epitopes. Another possibility is that there are two (or more) essential receptor-binding sites, so that obstructing one site is sufficient to prevent virus attachment to cells. Alternatively, one or both antibodies may bind at an allosteric site, triggering a structural change in another region of the spike which disrupts access to the receptor. However, root-mean-square deviations (RMSD) between Spike 8 with and without antibodies are low at 0.241 Å when 3E8 is bound and at 0.325 Å when 2D9 is bound, suggesting that the structural integrity of the spike is preserved upon antibody binding. Finally, the receptor-binding site may be in different places on spikes from different serotypes, but the observation that antibodies can target the spikes from two serotypes at the same epitope provides evidence against this argument. Efforts in our labs to discover the identity of the receptor and uncover the mechanism by which human astrovirus gains entry into cells are ongoing.

Secondly, the significant overlap between the epitope targeted by PL-2 on Spike 2 and the epitope targeted by 3E8 on Spike 8 shows that antibodies can target the same epitope on spikes from two HAstV serotypes and neutralize the virus by the same mechanism: by blocking attachment to cells. This epitope intersection indicates that functional regions on the spike surface are likely conserved across HAstV serotypes, despite a high degree of sequence variability in the spike, which has 41% to 76% sequence identity across all 8 classical serotypes (7).

Finally, our structures of scFv 3E8/Spike 8 and scFv 2D9/Spike 8 illustrate that the immune response can target the spike of a single serotype at different epitopes. This dual-targeting mechanism reveals that spikes used as immunogens elicit a neutralizing polyclonal response to human astrovirus. A polyclonal response is beneficial for generating long-term protection against infection because it minimizes virus neutralization escape by mutations. Indeed, the HAstV8 escape mutant to 3E8 is still susceptible to neutralization by 2D9 and vice versa (43). Therefore, immunization with spikes presents a robust strategy for HAstV subunit vaccine immunogen design. Importantly, these results also indicate that obstructing any of the multiple important sites on the spike surface can prevent virus infectivity. Consequently, HAstV has several weak points that could be exploited either individually or in combination with an antiviral therapy.

In conclusion, this work has increased our understanding of the antigenic structure of the human astrovirus capsid spike, the basis of virus neutralization by antibodies targeting the spike, and the functional sites on spike that are relevant for virus attachment to the host cell, thus supporting the development of vaccines and therapies against astrovirus gastroenteritis.

## MATERIALS AND METHODS

### Expression and purification of scFv 3E8.

Mouse hybridoma cells producing MAb 3E8 were generated as reported previously (43). The amino acid sequences of the MAb 3E8 variable regions were identified as described previously (48), allowing for recombinant antibody expression. A synthetic gene, codon optimized for Drosophila melanogaster and containing a 3E8 kappa chain variable region connected to the 3E8 heavy chain variable region by a GGS(GGGGS)_3_ linker, was purchased from Integrated DNA Technologies. This gene was cloned into the pCMV-VRC01 vector by Gibson Assembly in frame with an N-terminal secretion signal sequence and a C-terminal thrombin cleavage site followed by a Twin Strep-tag. The resulting pCMV-VRC01_scFv_3E8 expression plasmid was used to electroporate Chinese hamster ovary suspension (CHO-S) cells using the MaxCyte system. The scFv 3E8 was expressed for 8 days by CHO-S cells growing in CD OptiCHO expression medium (Gibco) supplemented with 1 mM sodium butyrate (Sigma-Aldrich), 8 mM l-glutamine (Gibco), 1× HT Supplement (Gibco), and 0.1% Pluronic F68 (Gibco) at 32°C with shaking at 125 rpm. Every 24 h, cells were fed with CHO CD EfficientFeed A (Gibco) supplemented with 7 mM l-glutamine (Gibco), 5.5% glucose (Sigma-Aldrich), and 23.4 g/liter yeastolate (BD). After 8 days, the cells were pelleted and medium containing secreted scFv 3E8 was 0.22-μm filtered, buffered to Strep wash buffer (50 mM Tris-HCl [pH 7.5], 150 mM NaCl, 1 mM EDTA), and supplemented with BioLock (IBA Lifesciences) to mask free biotin in the medium. The sample was 0.22-μm filtered again, affinity-purified on two tandem 5-ml StrepTrap HP columns (GE) and eluted in Strep elution buffer (Strep wash buffer with 2.5 mM desthiobiotin). The scFv 3E8 was dialyzed overnight into Tris-buffered saline (TBS; 10 mM Tris-HCl [pH 7.5], 150 mM NaCl). It should be noted that scFv expression in CHO-S cells was faster, more reliable, and yielded more protein than scFv expression in S2 cells.

### Expression and purification of scFv 2D9.

Mouse hybridoma cells producing MAb 2D9 were generated as reported previously (43). The amino acid sequences of the MAb 2D9 variable regions were identified as described previously (48), allowing for recombinant antibody expression. A synthetic gene, codon optimized for Drosophila melanogaster, containing a 2D9 kappa chain variable region connected to the 2D9 heavy chain variable region by a GGS(GGGGS)_3_ linker and flanked by BglII and NheI restriction sites, was purchased from Integrated DNA Technologies. This gene was cloned into the pMT_puro_BiP vector by restriction digestion in frame with an N-terminal BiP secretion signal sequence and a C-terminal thrombin cleavage site, followed by a Twin Strep-tag. The plasmid contained a metallothionein promoter for induction of gene expression as well as a puromycin-resistance gene. The resulting pMT_puro_BiP_scFv_2D9 expression plasmid was used to obtain stably transfected D. melanogaster Schneider 2 (S2) cells, by transfection with FuGENE HD (Promega) followed by selection with 5 μg/ml puromycin. The S2 cells were grown in Schneider’s S2 medium (Gibco) with 10% heat-inactivated fetal bovine serum and 1× Pen-Strep (Gibco) at the selection stage. Cells were then adapted to serum-free, antibiotic-free ESF 921 medium (Expression Systems) for expression. The stable S2 cells were grown in shaker flasks to a concentration of 3.0 × 10^6^ cells/ml. Expression of scFv 2D9 was induced with 500 μM CuCl_2_, and cells were incubated at 27°C with shaking at 125 rpm. After 5 days, the cells were pelleted and medium containing secreted scFv 2D9 was 0.22-μm filtered, buffered to Strep wash buffer (50 mM Tris-HCl [pH 8.0], 150 mM NaCl, 1 mM EDTA) and concentrated 200-fold by tangential flow filtration. After supplementation with BioLock (IBA Lifesciences) to mask free biotin in the medium, the sample was 0.22-μm filtered again, affinity purified on two tandem 5-ml StrepTrap HP columns (GE), and eluted in Strep elution buffer (Strep wash buffer with 2.5 mM desthiobiotin). The scFv 2D9 was dialyzed overnight into TBS (10 mM Tris-HCl [pH 8.0], 150 mM NaCl).

### Expression and purification of the Spike 8 antigen.

A synthetic gene, codon optimized for E. coli expression and encoding the HAstV serotype 8 capsid spike protein amino acids 429 to 647 (Spike 8, UniProtKB entry Q9IFX1), was purchased from Integrated DNA Technologies. To make the Spike 8 expression plasmid, the gene was cloned into pET52b (Addgene) under the control of the T7 promoter in frame with a C-terminal thrombin cleavage site and a 10-histidine purification tag. The plasmid was verified by DNA sequencing. Next, the plasmid was transformed into E. coli strain BL21(DE3). Cultures were inoculated and grown in LB/ampicillin medium. At an optical density of 0.6, protein production was induced with 1 mM isopropyl-d-thiogalactopyranoside (IPTG) at 18°C for 18 h. E. coli cells were lysed by ultrasonication in Buffer A (20 mM Tris-HCl [pH 8.0], 500 mM NaCl, 20 mM imidazole) containing 2 mM MgCl_2_, 0.0125 U/μl benzonase (Merck Millipore), and 1× protease inhibitor cocktail set V EDTA-free (Merck Millipore). The protein was batch-purified from soluble lysates with TALON metal affinity resin (GE) and eluted with Buffer A containing 500 mM imidazole. The protein was dialyzed overnight into TBS (10 mM Tris-HCl [pH 8.0], 150 mM NaCl) and further purified by size exclusion chromatography on a Superdex 75 column.

### Biolayer interferometry affinity determination of scFv 3E8 and scFv 2D9 for Spike 8.

His-tagged Spike 8 was diluted to 0.5 μg/ml in BLI blocking buffer (PBS [pH 7.4], 2% bovine serum albumin [BSA], 0.09% Tween 20). The scFv 3E8 was diluted to 80 nM (2.15 μg/ml) in BLI blocking buffer and five serial 1:2 dilutions were prepared. The scFv 2D9 was diluted to 20 nM (0.592 μg/ml) in BLI blocking buffer and five serial 1:2 dilutions were prepared. Using the 8-channel setting on an Octet RED384 instrument (FortéBio), pre-equilibrated Anti-Penta-His (HIS1K) sensor tips were dipped into the following solutions at 22°C with shaking at 1,000 rpm: First Baseline, BLI blocking buffer for 60 s; Loading, 0.5 μg/ml Spike 8 for 180 s; Second Baseline, BLI blocking buffer for 60 s; Association, 6 different concentrations of the scFv analyte in a serial 1:2 dilution for 60 s (scFv 3E8) or 120 s (scFv 2D9); and Dissociation, BLI blocking buffer for 120 s (scFv 3E8) or 180 s (scFv 2D9). A reference sample control was included in which 0 nM scFv was tested. A reference sensor control was also included, in which either 80 nM scFv 3E8 or 20 nM scFv 2D9 was associated to sensor tips that had not been loaded with Spike 8, to test for nonspecific scFv binding to the sensors. The sensors were dipped into BLI blocking buffer in these controls.

Each affinity determination experiment was performed in triplicate as independent assays. Data were processed separately and fit using the Octet Data Analysis software v.7 (FortéBio). Before fitting, all data sets were reference-subtracted, aligned to the baseline, and aligned for inter-step correction through their respective dissociation steps as per the manufacturer’s instructions. For each experiment, six different scFv analyte concentrations were used to fit association and dissociation globally using a 1:1 binding model. Ultimately, the goodness of fit was determined using *R*^2^ and χ^2^ values according to the manufacturer’s guidelines. The reported K_D_, *R*^2^, and χ^2^ values were averaged manually from the triplicate assays, generating the reported K_D_ standard deviation.

For the BLI competition assay, His-tagged Spike 8 was diluted to 0.5 μg/ml in BLI blocking buffer. The scFv 2D9 was diluted to 25 nM (0.74 μg/ml) in BLI blocking buffer. The scFv 3E8 was diluted to 400 nM (12 μg/ml) in BLI blocking buffer and used to prepare two serial 1:2 dilutions, followed by one serial 1:4 dilution, to obtain concentrations of 400, 200, 100, and 25 nM scFv 3E8. 0 nM scFv 3E8 was also tested. Pre-equilibrated Anti-Penta-His (HIS1K) sensor tips were dipped into the following solutions at 22°C with shaking at 1,000 rpm: First Baseline, BLI blocking buffer for 60 s; Loading, 0.5 μg/ml Spike 8 for 180 s; Second Baseline, BLI blocking buffer for 60 s; scFv 2D9 Association, 25 nM scFv 2D9 for 120 s; scFv 3E8 Association, 5 different concentrations of scFv 3E8 for 120 s; and Dissociation, BLI blocking buffer for 180 s. A reference sensor control was included in which 25 nM scFv 2D9, followed by 400 nM scFv 3E8, was associated to sensor tips that had not been loaded with Spike 8 to test for nonspecific scFv binding to the sensors. The sensors were dipped into BLI blocking buffer in this control. The data set was reference-subtracted and aligned to the scFv 3E8 association step to generate Fig. 1C.

### Formation and structure determination of the scFv 3E8/Spike 8 complex.

Complex formation was performed by incubating 3 molar excess scFv 3E8 with Spike 8 overnight at 4°C in TBS (pH 7.2). Simultaneously, the purification tags from both scFv 3E8 and Spike 8 were removed by digesting the complex with thrombin protease. The scFv 3E8/Spike 8 complex was purified by size exclusion chromatography on a Superdex 200 column in TBS (pH 7.2). The complex coeluted at an apparent molecular mass of ∼105 kDa compared to gel filtration standards, consistent with a 2:2 scFv 3E8:Spike 8 complex in solution (data not shown). The purified complex was concentrated to 6.33 mg/ml. Hanging drops (2 μl) were formed by a 1:1 addition of concentrated protein complex with a well solution of 0.2 M lithium citrate tribasic and 18% PEG 3350. Crystals were grown by hanging drop vapor diffusion at 22°C. The crystals then were transferred into a cryoprotectant solution of 0.2 M lithium citrate tribasic, 18.9% PEG 3350, and 25% glycerol and flash frozen in liquid nitrogen. Diffraction data from a single crystal were collected at cryogenic temperature at the Advanced Photon Source on beamline 23-ID-D using a wavelength of 1.033184 Å. The data were processed with Mosflm (ccp4i) and scaled with Aimless (ccp4i). The structure was solved by molecular replacement using Phenix.

### Formation and structure determination of the scFv 2D9/Spike 8 complex.

Complex formation was performed by incubating 3.5 molar excess scFv 2D9 with Spike 8 overnight at 4°C in TBS (pH 8.0). Simultaneously, the purification tags from both scFv 2D9 and Spike 8 were removed by digesting the complex with thrombin protease. The scFv 2D9/Spike 8 complex was purified by size exclusion chromatography on a Superdex 200 column in TBS (pH 8.0). The complex coeluted at an apparent molecular mass of ∼105 kDa compared to gel filtration standards, consistent with a 2:2 scFv 2D9:Spike 8 complex in solution (data not shown). The purified complex was concentrated to 3 mg/ml. Hanging drops (2 μl) were formed by a 1:1 addition of concentrated protein complex with a well solution of 0.1 M ammonium acetate, 0.1 M Bis-Tris (pH 5.5), and 16% PEG 10000. Crystals were grown by hanging-drop vapor diffusion at 22°C. The crystals were then transferred into a cryoprotectant solution of 0.1 M ammonium acetate, 0.1 M Bis-Tris (pH 5.5), 16.8% PEG 10,000, and 25% ethylene glycol and flash frozen in liquid nitrogen. Six diffraction data sets from a single crystal were collected at cryogenic temperature at the Advanced Light Source on Beamline 8.3.1 using a wavelength of 1.115830 Å. The six data sets were processed separately with XDS and scaled together with Aimless (ccp4i). The structure was solved by molecular replacement using Phenix.

### HAstV8 attachment inhibition assay.

Serial 1:5 dilutions of the ascites fluids for 3E8 or 2D9 were pre-incubated with infectious HAstV8 particles (multiplicity of infection [MOI] = 30) for 1 h at room temperature. Caco-2 cell monolayers grown in 48-well plates were washed once with PBS, and then blocking solution (1% BSA in PBS) was added for 45 min at room temperature, followed by a 15 min incubation on ice. The cells were then washed once with ice-cold PBS and incubated with the virus-antibody complex for 1 h on ice. MAb 3B4 against HAstV1 was used as a negative control. The unbound virus was washed three times with cold PBS, and the total RNA was extracted with TRIzol Reagent (Invitrogen) according to the manufacturer’s instructions. Viral RNA or cellular 18S RNA was reverse transcribed using MMLV reverse transcriptase (Invitrogen). RT-qPCR was performed with the premixed reagent Real Q Plus Master Mix Green (Amplicon), and the PCR was carried out in an ABI Prism 7500 Detection System (Applied Biosystems). The primers used to detect HAstV8 RNA were forward primer 5′ ATGAATTATTTTGATACTGAGGAAGATTACTTGGAA 3′ and reverse primer 5′ CTTTCTTGAGAAATAGATACCAAAGTACTTCAG 3′ (ORF 1b). For normalization, 18S ribosomal cellular RNA was amplified and quantified using forward primer 5′ CGAAAGCATTTGCCAAGAAT 3′ and reverse primer 5′ GCATCGTTTATGGTCGGAAC 3′. The arithmetic means and standard deviation error bars from four independent experiments, performed in duplicate (3E8 and 2D9) or as single replicas (3B4), are shown. A Mann-Whitney U test was used to compare samples with MAbs 3E8 or 2D9 to samples with control MAb 3B4.

### HAstV8 detachment assay.

Confluent Caco-2 cell monolayers in 48-well plates were blocked with 1% BSA in PBS for 45 min at room temperature followed by a 15 min incubation on ice. Purified HAstV8 viral particles were added at an MOI of 30 and then incubated for 1 h on ice to allow binding of the virus to the cell surface. The unbound virus was subsequently removed by washing three times with cold PBS. Serial 1:5 dilutions of the indicated ascites fluids of 3E8 and 2D9 were added to the cells and then incubated for 1 h on ice. After this incubation, the antibody and detached virus were removed with cold PBS, and RNA extraction and RT-qPCR quantification were performed as described above. MAb 3B4 against HAstV1 was used as a negative control. The arithmetic means and standard deviation error bars from three independent experiments, performed in duplicate (3E8 and 2D9) or as single replicas (3B4), are shown. A Mann-Whitney U test was used to compare samples with MAbs 3E8 or 2D9 to samples with control MAb 3B4.

### Expression and purification of chimeric Fabs 3E8, 2D9, and 3B4.

Synthetic cDNA encoding the kappa and heavy chain variable regions of each antibody was cloned by Gibson Assembly into the pCMV-VRC01 antibody vectors for light and heavy chains, in place of the variable regions of antibody VRC01, a human anti-HIV antibody targeting the gp120 protein (49). For the Fab heavy chain, only the variable region followed by the constant heavy 1 region ending with residues DKKVEPKSC was included, followed by an AS linker, C-terminal thrombin cleavage site, and a Twin Strep-tag. Sequences were in frame with the N-terminal signal sequence. The resulting chimeric Fab expression plasmids pCMV-Fab_kappa and pCMV-Fab_heavy_VH+CH1 (where Fab is 3E8, 2D9, or 3B4) contained both the variable regions from the original mouse antibodies and the constant regions from the human IgG1 antibody under the control of the human cytomegalovirus promoter. The plasmids were verified by DNA sequencing. The expression plasmids were used at a 3:2 ratio of kappa chain: heavy VH + CH1 chain to electroporate Chinese Hamster Ovary suspension (CHO-S) cells using the MaxCyte system. Recombinant chimeric Fabs were expressed for either 9 days (Fabs 3E8 and 3B4) or 7 days (Fab 2D9) by CHO-S cells, which were grown in CD OptiCHO expression medium supplemented with 1 mM sodium butyrate, 8 mM l-glutamine, 1× HT supplement, and 0.1% Pluronic F68 at 32°C with 125-rpm shaking. Every 24 h, cells were fed with CHO CD EfficientFeed A supplemented with 7 mM l-glutamine, 5.5% glucose, and 23.4 g/liter yeastolate. After 9 days, the cells were pelleted and medium containing secreted Fabs was 0.22-μm filtered, buffered to Strep wash buffer (50 mM Tris [pH 7.5], 150 mM NaCl, 1 mM EDTA), and supplemented with BioLock (IBA Lifesciences) to mask free biotin in the medium. The samples were 0.22-μm filtered again, affinity purified on two tandem 5-ml StrepTrap HP columns (GE), and eluted in Strep elution buffer (Strep wash buffer with 2.5 mM desthiobiotin). Fabs 3E8 and 3B4 were dialyzed overnight into TBS (10 mM Tris-HCl [pH 7.2], 150 mM NaCl). Fab 2D9 was dialyzed overnight into 1× PBS (pH 7.4; Sigma-Aldrich).

### GFP-Spike 8 attachment inhibition assay by fluorescence microscopy.

GFP-Spike 8 was expressed and purified as described previously (42). 24-well plates containing fibronectin-treated glass coverslips were seeded with 100,000 Caco-2 cells per well and allowed to adhere overnight. 250-μl samples containing 400 nM GFP-Spike 8 were incubated with 3 molar excess (1,200 nM) of antigen-binding fragments (Fabs) of the Spike 8-specific monoclonal antibodies 3E8 and 2D9 and the Fab of the Spike 1-specific monoclonal antibody 3B4 for 1 h at room temperature in Dulbecco's phosphate-buffered saline (PBS; Gibco). Samples of PBS alone and 400 nM GFP were used as controls for autofluorescence and nonspecific binding, respectively. Media was aspirated from the cell monolayer, and then Spike-Fab mixtures were added to Caco-2 cells and incubated at 4°C for 1 h. Protein mixtures were removed, and cells were washed with PBS and fixed with 2% paraformaldehyde (Thermo Fisher) in PBS for 15 min. Cells were washed with PBS and then stained with Hoechst 33342 dye (Thermo Fisher) in PBS for 30 min. Coverslips were washed with PBS, dried, and mounted in Vectashield mounting media on glass microscope slides.

Z-stack images were acquired by using identical acquisition parameters with a Zeiss Axio Imager equipped with an AxioCam 506 monochrome camera using an oil-immersion 100×/1.4 NA Plan Apo objective lens. Z-stack images contained 9 slices at 0.24-μm intervals, with GFP and Hoechst channels exposed for 1,600 and 95 ms, respectively. GFP signal was collected with a Zeiss Fset38 filter cube, and Hoechst signal was collected with a Zeiss Fset49 filter cube. After acquisition, images were deconvolved with AutoQuant X 3D deconvolution software (Media Cybernetics Version X3.1.3) for 10 iterations, using the Z-montage option. After deconvolution, a median filter with a 2-pixel kernel size was applied to the GFP channel of all images to reduce noise. Linear histogram adjustments were made to the GFP channel using FIJI (50), such that minimum values were 1,300 and maximum values were 7,500, to help reduce background fluorescence. Single Z-stack slices from representative images were then converted to a single RGB image. Images were cropped to identical sizes in FIJI to select representative cells and are representative of data from at least three independent experiments.

### Data availability.

Coordinates and structure factors for the scFv 3E8/Spike 8 structure and the scFv 2D9/Spike 8 structure have been deposited in the Protein Data Bank under accession codes 7RK1 and 7RK2, respectively. All other data have been made available in the article.
